# Study of Electronic and Transport Properties in Double-Barrier Resonant Tunneling Systems

**DOI:** 10.3390/nano12101714

**Published:** 2022-05-17

**Authors:** John A. Gil-Corrales, Juan A. Vinasco, Miguel E. Mora-Ramos, Alvaro L. Morales, Carlos A. Duque

**Affiliations:** 1 Grupo de Materia Condensada-UdeA, Instituto de Física, Facultad de Ciencias Exactas y Naturales, Universidad de Antioquia UdeA, Calle 70 No. 52-21, Medellín 050022, Colombia; jalexander.gil@udea.edu.co (J.A.G.-C.); juan.vinascos@udea.edu.co (J.A.V.); carlos.duque1@udea.edu.co (C.A.D.); 2 Centro de Investigación en Ciencias-IICBA, Universidad Autónoma del Estado de Morelos, Av. Universidad 1001, Cuernavaca 62209, Morelos, Mexico; memora@uaem.mx; 3 Grupo de Estado Sólido, Instituto de Física, Facultad de Ciencias Exactas y Naturales, Universidad de Antioquia Udea, Calle 70 No. 52-21, Medellín 050022, Colombia

**Keywords:** resonant tunneling diode, electronic transmission probability, Landauer formalism

## Abstract

Resonant tunneling devices are still under study today due to their multiple applications in optoelectronics or logic circuits. In this work, we review an out-of-equilibrium GaAs/AlGaAs double-barrier resonant tunneling diode system, including the effect of donor density and external potentials in a self-consistent way. The calculation method uses the finite-element approach and the Landauer formalism. Quasi-stationary states, transmission probability, current density, cut-off frequency, and conductance are discussed considering variations in the donor density and the width of the central well. For all arrangements, the appearance of negative differential resistance (NDR) is evident, which is a fundamental characteristic of practical applications in devices. Finally, a comparison of the simulation with an experimental double-barrier system based on InGaAs with AlAs barriers reported in the literature has been obtained, evidencing the position and magnitude of the resonance peak in the current correctly.

## 1. Introduction

Resonant tunneling diodes (RTDs) are semiconductor devices that consist of a system of two or more potential barriers that allow electron transport only for certain states known as resonant states. The operating mechanism is fundamentally based on the tunneling effect of quantum mechanics. This type of system is characterized by developing one or more NDR zones that are the fundamental peculiarity of RTDs that enable them to be used for various applications. These devices are experimentally developed in very thin layers, which allows for an ultrafast operation speed, enabling them for applications even in the terahertz range [1,2,3,4]. The first investigations in the field of resonant tunneling were carried out around 50 years ago; some of these early developments are included in the references [5,6,7,8,9,10,11,12]. These studies have progressed continuously, characterizing RTDs both experimentally and theoretically. To mention some of the most recent work in this area, Citro and Romeo [13] studied a flux-tunable tunneling diode using a mesoscopic ring subject to the Rashba spin–orbit interaction and sequentially coupled to an interacting quantum dot, in the presence of an Aharonov–Bohm flux. Wójcik et al. [14] studied the spin- and time-dependent electron transport in a paramagnetic resonant tunneling diode using the self-consistent Wigner–Poisson method. They found that under a constant bias, both the spin-up and spin-down current components exhibit the THz oscillations in two different bias voltage regimes. In more recent works, Shinkawa et al. [15] studied the hole-tunneling in Si${}_{0.82}$ Ge${}_{0.18}$/Si asymmetric-double-quantum-well RTD with high-resonance current and suppressed thermionic emission. Simultaneously, Encomendero et al. [16] investigated the possibility of using degenerately doped contact layers to screen the built-in polarization fields and recover symmetric resonant injection; they found negative differential conductance (NDC) under both bias polarities of GaN/AlN RTDs. Several theoretical works that approach resonant tunneling systems by means of the non-equilibrium Green’s formalism (NEGF) for the calculation of the transmission function have been reported [17,18].

As an application of tunneling systems, Belkadi et al. [19] demonstrated the appearance of resonant tunneling effects in metal-double-insulator-metal-type diodes, by varying the thickness of insulators to modify the depth and width of the quantum well. Various analytical works [20,21,22] have sought an improvement in the efficiency, a decrease in the dissipation power, and the optimization of parameters to obtain the highest peak-to-valley current ratio in resonant tunneling systems of various materials, which can improve the applicability in practical devices. Finally, in recent reports [23,24], resonant systems are evidenced as possible direct applications in communication systems for frequencies of the order of Gigahertz or the possibility of using RTDs as memory systems. Among the experimental developments in this area, in works such as those from Ryu and co-workers [25],a semitransparent cathode of indium tin oxide (ITO)/Ag/ITO is studied, developed as a resonant tunneling double-barrier structure for transparent organic light-emitting diodes. This was achieved by employing an e-beam evaporated ITO/Ag/ITO cathode due to the double quantum barriers of ITO and the quantum well of Ag. Their results include the observation of a weak NDR in devices using a 100 nm thick ITO/Ag/ITO layer as a cathode in combination with a thin LiF/Al layer. In a later work, Ryu et al. [26] developed a semitransparent multilayered cathode of indium tin oxide (ITO)/Ag/tungsten oxide (WO${}_{3}$) for transparent organic light-emitting diodes (LEDs). The device showed a weak NDR, until the operating voltage of 8 V was reached.

It is worth mentioning some recent experimental and theoretical related works that have sought to improve the efficiency of optoelectronic devices, including perovskite light-emitting diodes (PeLEDs) [27], solar cells that use resonant semiconductor nanoparticles (NPs) and improve both light trapping and scattering [28], multiple quantum well (MQW), and quantum dot (QD) nitride-based light-emitting diodes (LEDs) [29,30].

Over the years, knowledge about the operation and physics behind this type of semiconductor devices has expanded, thus allowing numerous applications, among which we can highlight: Wei and Shen’s work, where a novel universal threshold logic gates (UTLG) based on RTD with simple structure and fixed parameters are proposed, taking advantage of the characteristics of NDR [31]. Jijun et al. analyzed the piezoelectric effects in RTDs based on GaAs/In${}_{x}$Ga${}_{1-x}$As/AlAs for potential applications in micromachined mechanical sensors, obtaining the result that the piezoresistive sensitivity of RTDs can be adjusted through the bias voltage [32]. Due to their particular NDR behavior, these systems are excellent candidates for applications to nanoelectronics; in this sense, Malindretos et al. grew a GaAs/AlAs RTD by means of a molecular beam epitaxy; their results are satisfactory and of good precision to fabricate RTDs suitable for application in robust digital logic circuits [33]. Among all the applications of this type of system, it is worth highlighting applications in detector devices that can filter in a varied range of frequencies by simply modifying geometric or material parameters. To mention such a device, Dong et al. [34] developed an RTD based on In${}_{0.53}$Ga${}_{0.47}$. As for detection in the 1550 nm range, they found that the detector responsivity is nonconstant, it decreases with the increase of the incident light power density, which provides the basis for optimizing the RTD performance.

Resonant tunneling systems go beyond double-barrier-based systems. In 2020, Mehmet Bati [35] studied the effects of an intense laser field on the properties of resonant tunneling in a double-well-structure parabolic reverse triple-barrier system, implementing the method of finite differences combined with the Green function formalism to calculate the transmission functions, obtaining the conclusion that the increment of the well width causes the incident electron waves to be localized. Consequently, the transmittance decreases, and the resonant peak becomes small or disappears.

In this problem, the Landauer approach has been chosen for the calculation of conductance, since it is a model that has a fairly broad theoretical development and that has been studied in depth for more than 40 years. In 1981, Langreth D.C. and Abrahams E. presented a rigorous derivation of the conductance formula from the linear response theory (Kubo’s formula), giving a generalization to the case of many-scattering-channel and found that only in very special circumstances can the currents in different channels be decoupled in such a way as to give a simple conductance formula [36]. Six years later, S. Eränen and J. Sinkkonen further generalized this formalism, studying the electrical current transport in conductor–insulator–conductor structures, where the charge carriers are assumed to traverse the insulating layer by tunneling. They self-consistently solved the coupled system of Poisson and Boltzmann equations, the latter giving the form of temporary relaxation. An important conclusion of this work is that the tunneling current density comes from the contribution of two effects: the first is the ordinary contribution of the Landauer formula in the linear voltage regime, and the second is a correction term originated by the screening of the electric potential through the insulating layer. The contribution to the current due to this screening term in most systems is negligible compared to the current generated by the effect of resonant tunneling. These results are detailed in [37].

Apart from the effect of the charge redistribution caused by the ionized donors and the external electric field applied to the contacts, it is possible to analyze the effect of the electronic spin dependence on the transport properties in magnetic RTD. The three combined effects generate an effective modification in the profile associated with the bottom of the conduction band in the heterostructure that finally causes modifications in the electronic transport properties. In the work of Havu et al. [38], the self-consistent spin-density-functional theory method was implemented within the Wigner formalism with Green functions to analyze the properties of electronic transport in a magnetic RTD obtaining the electronic densities and potentials, studying the computational cost that this requires.

Over the years, research has continued to improve numerical techniques to make them more efficient and extend theoretical developments in various physical situations. Taking advantage of the versatility in terms of materials and external parameters with which it is possible to develop RTDs, our main interest is to develop a methodological approach to address these types of problems by first solving the effect of charge redistribution and electron density in the system out of equilibrium to obtain in this way the profile at the bottom of the self-consistent conduction band. This will act as an input parameter for the potential term in the Schrödinger equation, considering open boundary conditions in the effective mass approximation, this equation, as well as the Poisson equation, is solved through the finite-element method (FEM) to obtain a set of quasi-stationary states and probabilities of electronic transmission in the system. Finally, with these transmission functions, the Landauer formalism is implemented for the calculation of the density current and conductance. In this study, we report the self-consistent potentials, quasi-stationary electronic states, tunneling currents, and conductances for different widths of the central well and different donor densities; then, a comparison is drawn between theoretical results of this procedure with recently reported experimental results. The article is organized as follows: Section 2 presents the theoretical framework of the simulation. In Section 3, we show and discuss the results obtained. Section 4 is devoted to the conclusions of the work.

## 2. Theoretical Model

**Figure 1:**
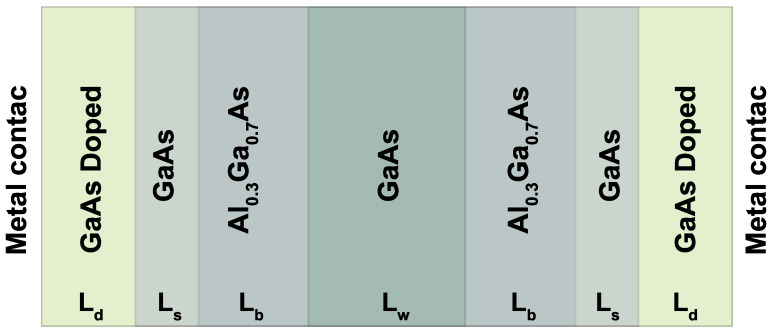
Scheme of the resonant tunneling diode (RTD), with doping $n_{d}$ in the outer regions, two Al${}_{0.3}$Ga${}_{0.7}$As barriers, a GaAs well, and two outer regions of the GaAs undoped with two metal contacts in the external regions.

Our system corresponds to an RTD (Resonant Tunneling Diode), consisting of a GaAs central region (Quantum Well) of length $L_{w}$, surrounded by two Al${}_{0.3}$Ga${}_{0.7}$As barriers with equal lengths $L_{b}$. There are two additional GaAs undoped spacers with lengths $L_{s}$; the purpose of these layers is to prevent electronically tunneling scattering effects due to impurities in the contact region. Finally, two outer GaAs doped layers of length $L_{d}$. This is the central system that is contacted with two electronic reservoirs or metal contacts on each side, as presented in Figure 1. For this work, we consider a non-rectifying metal-semiconductor junction, which consists of a negligible relative resistance of the contacts compared to the resistance of the central device. In this case, it is considered that mobility effects are due solely to the electronic movement in the conduction band.

The system was solved through the finite-element method with the COMSOL- Multiphysics licensed software by (5.4, COMSOL AB, Stockholm, Sweden) [39,40,41], implementing the semiconductor module (“Semiconductor Module User’s Guide” COMSOL Multiphysics^®^) [42]. As a starting point, we must consider the effect on the potential of the donor density and electron density in the system—this can be modeled by means of the Poisson equation,

(1) $$\vec{\nabla }\cdot \left(\epsilon_{0}\epsilon_{r}\vec{\nabla }V\left(x\right)\right)=-\rho \left(x\right),$$

in this equation, $\epsilon_{r}$ and $\epsilon_{0}$ are the relative permittivity and vacuum permittivity, respectively, and the charge density ρ(x) has the form

(2) $$\rho \left(x\right)=-q_{e}\left(n\left(x\right)-N_{d}\right),$$

in this equation, $N_{d}$ is the number of donors which are considered to be fully ionized, and $q_{e}$ is the electronic charge. The electronic density has the form

(3) $$n\left(x\right)=N_{c}\gamma_{n}e^{-\beta \;(E_{c}-E_{F})},$$

in Equation (3), $E_{c}$ is the bottom of the conduction band, which, due to the effect of the redistribution of charges, is not a straight line, but it is a function that can vary with position, $E_{F}$ is the quasi-Fermi level (for the system out of equilibrium) associated with the conduction band. The term $Nc=2{\left(m^{*}/\;2\pi \beta \hbar^{2}\right)}^{3/2}$ corresponds to the effective density of states, with $m^{*}$—the electron effective mass, $k_{B}$ is the Boltzmann constant, *ℏ* is the reduced Planck constant, and β is the Boltzmann factor $\beta =1/k_{B}T$. In Equation (3), the term $\gamma_{n}$ is equal to

(4) $$\gamma_{n}=F_{1/2}\left(\beta (E_{F}-E_{c})\right)e^{\beta (E_{c}-E_{F})},$$

where $F_{1/2}$ is the Fermi–Dirac integral, $k_{B}$ is the Boltzmann constant, and *T*—the system temperature. In the non-degenerate states limit, the Fermi–Dirac distribution approaches the Maxwell–Boltzmann distribution, and $\gamma_{n}$ = 1. The values of the electronic affinity $q_{e}\;\chi =E_{0}-E_{c}$ ($E_{0}$ is the vacuum level) and the bandgap $E_{g}=E_{c}-E_{v}$ are input parameters associated with the properties of the materials and necessary to establish the quasi-Fermi level as a reference point during the calculations. The band energies in Equations (3) and (4) are related to the electrostatic potential V(x), as follows [43,44]:

(5) $$E_{c}=-\chi -q_{e}\;V\left(x\right).$$

The potential obtained in Equation (5) can be replaced in the Schrödinger equation to obtain the eigenfunctions and eigenvalues,

(6) $$-\hbar^{2}\vec{\nabla }\cdot \left(\frac{\vec{\nabla }\psi_{i}\left(\vec{r}\right)}{2m^{*}}\right)+U\left(\vec{r}\right)\psi_{i}\left(\vec{r}\right)=E_{i}\psi_{i}\left(\vec{r}\right),$$

**Figure 2:**
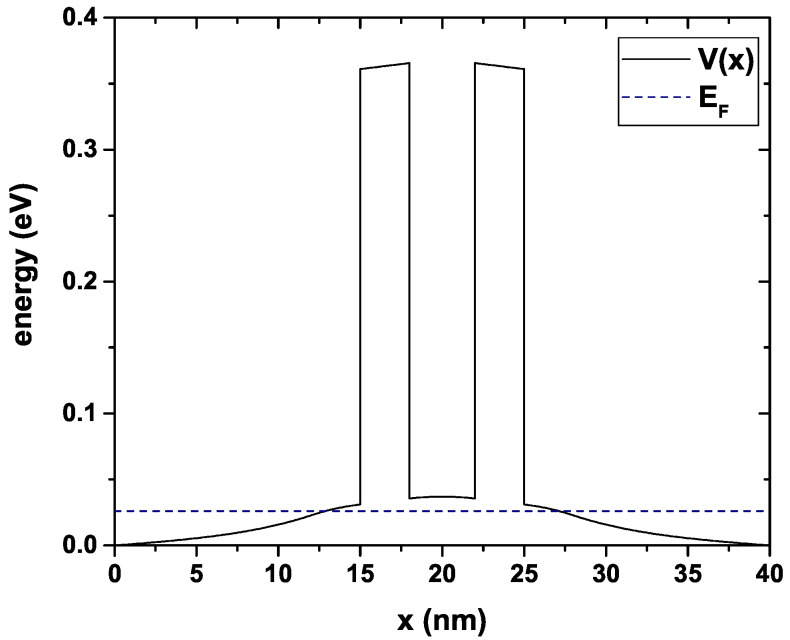
Conduction band profile. The dashed line correspond to the quasi-Fermi Level. The calculations are for $L_{w}=4$ nm, $L_{b}=3$ nm, $L_{s}=3$ nm, $L_{d}=12$ nm and $n_{d}=1.2\times 10^{18}$ cm${}^{-3}$.

*ℏ* is the reduced Planck constant, $U\left(\vec{r}\right)=E_{c}$ is the conduction band profile obtained by solving the system of Equations (1)–(5) in a self-consistent way, this includes the potential band offset and the effect of the charge redistribution due to doping. $E_{c}$ is depicted in Figure 2 for $L_{w}$ = 4 nm, $L_{b}$ = 3 nm, $L_{s}$ = 3 nm, $L_{d}$ = 12 nm and $n_{d}$ = 1.2$\;\times \;10^{18}$ cm${}^{-3}$, the quasi-Fermi level calculated for this configuration is $E_{F}$ = 0.026 eV, as presented in Figure 2 with the dashed line. $\psi_{i}$ is the system wave function corresponding to the eigenvalue $E_{i}$, the subscript *i* indicates the quasi-stationary states generated inside the device central quantum well region. The solutions of Equation (6) for this system considering open boundary conditions are plane waves,

(7) $$\psi_{i}\left(\vec{r}\right)=A\left(\vec{r}\right)e^{i{\vec{k}}_{i}\cdot \vec{r}}+B\left(\vec{r}\right)e^{-i{\vec{k}}_{i}\cdot \vec{r}}.$$

The functions $A(\vec{r})$ and $B(\vec{r})$ indicate that the amplitude of the wave that propagates from left to right and from right to left depends directly on the point in the system at which they are calculated and the full wave function is a superposition of these waves, $k_{i}$ is the magnitude of the wave vector and is given by $k_{i}={\left(2m^{*}\left(E_{i}-E_{c}\right)/\hbar^{2}\right)}^{1/2}$.

Knowing the amplitude of the wave function in all regions, it is possible to calculate the transmission function T(E) through the device by

(8) $$T\left(E\right)=\frac{|A\left({\vec{r}}_{f}\right){|}^{2}}{|A\left({\vec{r}}_{i}\right){|}^{2}},$$

where $A({\vec{r}}_{i})$ represents the wave amplitude that propagates from left to right evaluated at the emitter (amplitude of the incident wave), and $A({\vec{r}}_{f})$ is the amplitude of a wave that propagates from left to right, but is evaluated in the collector (amplitude of the transmitted wave). This function is proportional to the probability of electronic tunneling through the double-barrier system.

Current-voltage characteristics of this device can be calculated using the Landauer formula, which gives the electronic tunneling current between the contacts,

(9) $$I=\frac{e}{\pi \hbar }\int_{-\infty }^{\infty }T\left(E\right)\left[F_{em}\left(E,\Phi \right)-F_{col}\left(E,\Phi \right)\right]dE,$$

in this equation, *e* is the electron charge and *ℏ* is the reduced Plank constant, the terms $F_{em}\left(E,\Phi \right)$ and $F_{col}\left(E,\Phi \right)$ correspond to the Fermi functions evaluated at the emitter and collector, respectively, given by $F_{em}\left(E,\Phi \right)={\left(1+e^{\left(E-E_{F}\right)/k_{B}T}\right)}^{-1}$ and $F_{col}\left(E,\Phi \right)={\left(1+e^{\left(E-\left(E_{F}-\Phi \right)\right)/k_{B}T}\right)}^{-1}$, where the term Φ is the bias voltage applied between both device terminals.

At the zero bias limit (at low temperature), the Fermi functions take the form of Heaviside functions and the Equation (9) reduces to the well-known Landauer equation for conductance [45],

(10) $$G=\frac{e^{2}}{\pi \hbar }\;T\left(E\right),$$

the term T(E) represents the transmission function between the contacts, which, in this case, corresponds to the two terminals of the device (emitter and collector).

### 2.1. A Device Macroscopically Large in the Transverse Directions

In most of these types of devices, it is reasonable to consider that the structure growth direction is very small compared to the transverse directions of the device. Considering this statement, the electronic energy associated with their transverse directions is given by

(11) $$\varepsilon_{y,z}=\frac{\hbar^{2}}{2m^{*}}\left(k_{z}^{2}+k_{y}^{2}\right).$$

With this expression, it is possible to calculate the electronic distribution, depending only on the device growth direction, obtaining,

(12) $$F\left(E\right)=S\;\frac{m^{*}}{\beta \pi \hbar^{2}}\;ln\left(1+e^{\beta (E_{F}-E)}\right),$$

where *S* is the cross-sectional area of the device and $\beta =1/k_{B}T$, with $k_{B}$—the Boltzmann constant and *T*—the device temperature. This expression is proportional to the number of electrons with energy *E*. With these results, it is possible to calculate the total current density through the device J=I/S, using Equation (9),

(13) $$J=\frac{em^{*}}{2\beta \pi^{2}\hbar^{3}}\int_{0}^{\infty }T\left(E\right)\;ln\left(\frac{1+e^{\beta (E_{F}-E)}}{1+e^{\beta (E_{F}-E-\Phi )}}\right)dE.$$

By means of this equation, it is possible to calculate the current-voltage characteristics through the device, considering variations in electronic concentration and temperature. In this type of system, the electronic transport process is ballistic, that is, there are no dispersion mechanisms within the device; however, the current does not reach infinite values due to the electron reflection probability being different from zero, which acts as a resistance to the passage of charge carriers through the system [46].

**Figure 3:**
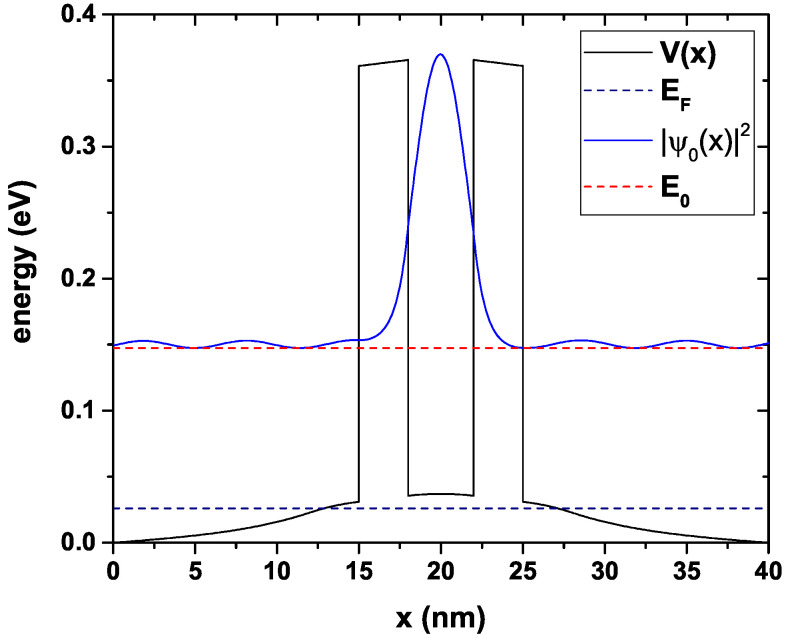
Potential energy for the system in equilibrium (bias voltage 0.0 V), the blue curve corresponds to the probability density of the resonant state, and the red dashed curve is the energy for this state $E_{0}$. The quasi-Fermi level is also presented with the blue dashed curve. The calculations are for $L_{w}=4$ nm, $L_{b}=3$ nm, $L_{s}=3$ nm, $L_{d}=12$ nm and $n_{d}=1.2\times 10^{18}$ cm${}^{-3}$.

In Figure 3, we can see the conduction band’s profile and the probability density that corresponds to the only quasi-stationary level within the central region. It is noteworthy that the energy corresponding to this level has an imaginary part, as it is expected for this type of confinement in which the electrons do not remain indefinitely inside the well, but they may eventually come out through tunneling through the walls of AlGaAs after a certain half-life that is proportional to the width of the transmission function peak, as will be seen later. Since the state presented in this figure corresponds to the quasi-stationary state of the central region (that is, the free electrons have exactly the same energy as the state inside the well), then it corresponds to a state of maximum tunneling probability, and therefore, the wave function amplitude is not affected after the electrons cross the two barriers. For any other free-electron energies in the emitter region, a decrease in the amplitude of the wave function occurs, which implies a decrease in the probability of transmission. The red dashed curve corresponds to the quasi-stationary state with energy $E_{0}$ = 0.147 eV; this is precisely the energy for which there must be a maximum electron tunneling incident from the emitter.

### 2.2. Cut-Off Frequency Calculation

One way to characterize RTDs is through the cut-off frequency. The impedance of unbiased RTDs is well described by an equivalent circuit (EC) consisting of parallel-connected resistance and capacitance and an additional resistance connected in series with this parallel combination [47,48,49]. To calculate the cut-off frequency, it is necessary to calculate the delay time of electrons in the quantum well of an RTD, $\tau_{D}$. This time can be calculated by remembering that the energies associated with the quasi-stationary states inside the central well are complex, E=ε+iΓ, where ε and Γ are the real part and the imaginary part of the energy, respectively. The imaginary component of energy can be associated with the $\tau_{D}$-time through the relation $\tau_{D}=\hbar /\Gamma$. In the case in which there is the contribution of two conduction channels with associated delay times $\tau_{1}$ and $\tau_{2}$, the total time $\tau_{D}$ is obtained according to $1/\tau_{D}=1/\tau_{1}+1/\tau_{2}$. We have calculated the cut-off frequency based on the work of Alkeev et al. [50], obtaining the result of

(14) $${\left(2\pi \;f_{C}\right)}^{2}=\frac{1}{\tau_{0}\;\tau_{D}}\;\left(\sqrt{1+A^{2}}-A\right),$$

with

(15) $$A=\frac{\tau_{0}^{2}+\tau_{D}^{2}}{2\;\tau_{0}\;\tau_{D}}.$$

In Equation (14), $f_{C}$ corresponds to the cut-off frequency and $\tau_{0}$ is a parameter that for this system takes estimated values between 0.1 ps and 0.2 ps, according to the results of Alkeev et al. [50].

## 3. Results and Discussion

For the transmission calculation, except for the conductance, the following input parameters have been used [51,52] at 300 K, for GaAs: electron effective mass $m^{*}=0.067\;m_{0}$ (where $m_{0}$ is the mass of the free electron), dielectric constant $\epsilon_{r}=12.9$, bandgap 1.42 eV, and electronic affinity χ=4.07 eV. For Al${}_{0.3}$Ga${}_{0.7}$As: electron effective mass $m^{*}=0.0879\;m_{0}$, dielectric constant $\epsilon_{r}=12.048$, bandgap 1.81 eV, and electronic affinity χ=3.74 eV. For the conductance calculations at 5 K, the following parameters have been used, for GaAs: electron effective mass $m^{*}=0.0665\;m_{0}$, dielectric constant $\epsilon_{r}=12.4$, and bandgap 1.52 eV. For Al${}_{0.3}$Ga${}_{0.7}$As: electron effective mass $m^{*}=0.0916\;m_{0}$, dielectric constant $\epsilon_{r}=11.56$, and bandgap 1.95 eV. All the equations were solved through the finite-element method considering the following parameters: 538 elements, 538 edge elements, 0.5149 element length radius, 400 as the maximum number of iterations of the self-consistent method, and $10^{-6}$ as the absolute tolerance.

**Figure 4:**
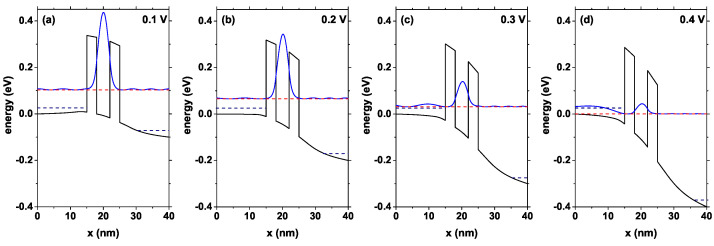
Potential energy change with bias voltage from 0.0 V to 0.4 V, the blue curve corresponds to the resonant state probability density and the red curve is the energy for this state $E_{0}$. The quasi-Fermi level is also presented by the dark blue dashed line for emitter and collector. The calculations are for $L_{w}=4$ nm, $L_{b}=3$ nm, $L_{s}=3$ nm, $L_{d}=12$ nm, and $n_{d}=1.2\times 10^{18}$ cm${}^{-3}$.

Figure 4 shows the self-consistent potential profile and how it changes and the resonant state probability density as the potential difference between the emitter and the collector increases. The clear redshift of the resonant level must be highlighted and the decrease in the electronic probability density inside the quantum well, mainly due to the electric field effect. For voltages higher than 0.4 V, the resonant state is very close to the bottom of the conduction band at the emitter, and therefore, from this limit, it no longer contributes to the transport properties in the system. From Figure 4d, it is possible to notice that, for high voltages, electronic current there will no longer exist in the system due to resonant tunneling, the incident electrons do not have a state inside the well to tunnel and therefore, their probability of passing to the collector must be significantly decreased. This occurs until reaching a certain limit voltage from which the electrons will have two options—perform resonant tunneling with a higher state of the system or perform non-resonant tunneling, which depends on both geometric characteristics and the materials involved in the system.

**Figure 5:**
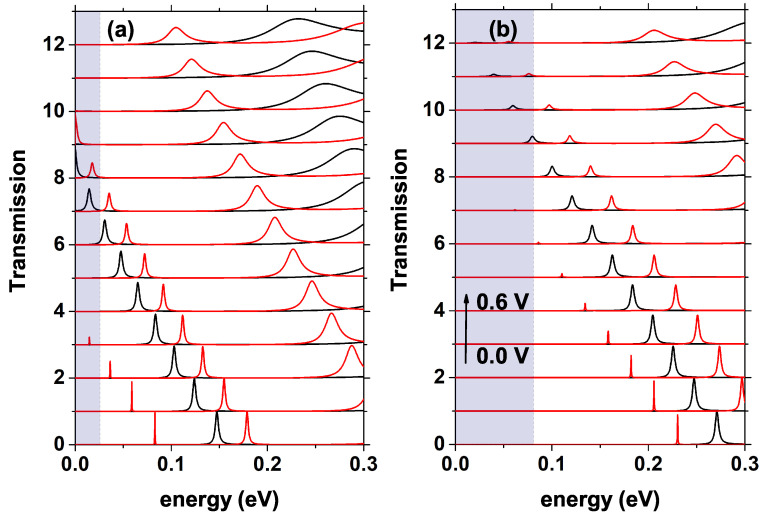
Transmission coefficient for different values of bias voltage, the black curve is for $L_{w}$ = 4 nm and, the red curve is for $L_{w}$ = 10 nm. (**a**) with $n_{d}$ fixed at 1.2$\;\times \;10^{18}$ [1/cm${}^{3}$] and (**b**) with $n_{d}$ fixed at 10$\;\times \;10^{18}$ [1/cm${}^{3}$]. The shaded area indicates the region between the bottom of the conduction band and the quasi-Fermi level at the emitter. As indicated by the arrow in (**b**), the voltage for each curve varies from 0.0 V to 0.6 V in steps of 0.05 V.

In Figure 5, we see the transmission probability for different voltage values, as indicated by the arrow, the voltage varies from 0.0 V to 0.6 V, Figure 5a corresponds to a doping $n_{d}$ = 1.2$\;\times \;10^{18}$ [1/cm${}^{3}$], while in Figure 5b it is $n_{d}$ = 10$\;\times \;10^{18}$ [1/cm${}^{3}$]. The red curve is for a 10 nm QW, and the black one is 4 nm. As indicated in Figure 5b, the quasi-Fermi level at the emitter (shaded region) presents a higher value for the system that has higher doping, which means that there is a greater number of occupied states in the conduction band that can contribute to current through the device. In Figure 5a, the quasi-Fermi level at the emitter takes the value of $E_{F}=0.026$ eV, while in Figure 5b, it is of $E_{F}=0.081$ eV. For zero voltage, the system with $L_{w}=4$ nm presents a single resonant level in 0.147 eV, while for $L_{w}=10$ nm, there are two resonant peaks in 0.083 eV and 0.179 eV, respectively, where the one closest to the bottom of the band has a medium amplitude lower than the peak of the highest quasi-steady state, this characteristic in the amplitudes of the peaks is maintained approximately, independent of the applied voltage. As the voltage is increased, as mentioned in Figure 4, there is a redshift of the levels inside the well and, at the same time, a decrease in the transmission amplitude; this behavior is clearly evidenced in Figure 5a,b. In Figure 5b, where the system presents higher doping, for $L_{w}=4$ nm now presents the resonant state for the energy of 0.271 eV, that is, 0.124 eV higher than in the case of lower donor density presented in Figure 5a. A fundamental difference concerning the system with lower $n_{d}$ is that now, for $L_{w}=10$ nm, there is only one resonant state inside the well and not two as occurs in the initial case, with an energy of 0.230 eV, which, as in Figure 5a, presents a much smaller mean amplitude than for the QW of $L_{w}=4$ nm.

In Figure 5, notice how as the voltage increases, the redshift of all the states occurs. For voltages higher than 0.05 V, the system with $L_{w}=10$ nm presents a third resonant peak well-defined of greater average width than the previous two; this does not happen for the system with $L_{w}=4$ nm. The increase in the average width of each peak occurs because the upper states are “less stable”, that is, the lifetime of the electrons in these states is less than in the lower states, and this time is proportional to the imaginary part of the energy associated with each of these states and the average width of the resonant peaks. The shaded area indicates the region between the bottom of the conduction band and the quasi-Fermi level at the emitter, as shown in Figure 5a; note how the first resonant peak reaches the quasi-Fermi level at the emitter faster for the system with less doping, at approximately 0.1 V for $L_{w}=10$ nm, and 0.3 V for $L_{w}=4$ nm. In the case of higher doping, these values become 0.3 V and 0.45 V for $L_{w}=10$ nm and $L_{w}=4$ nm, respectively. This indicates that the system in Figure 5a will reach a peak in the current faster than the system in Figure 5b.

**Figure 6:**
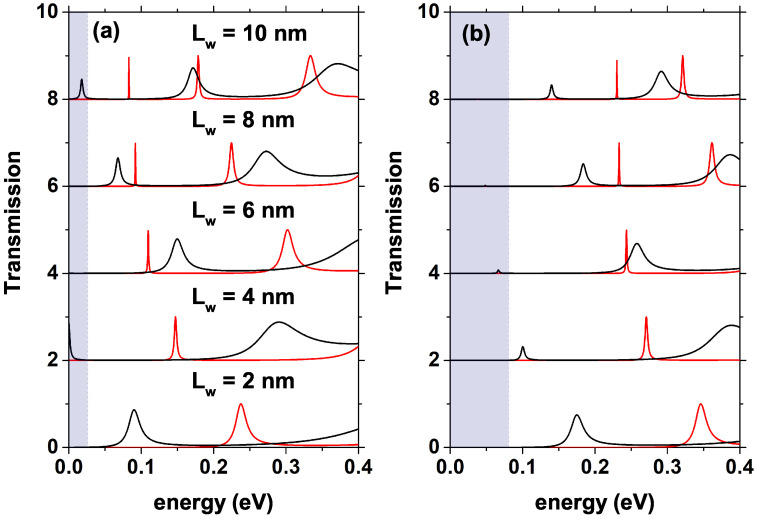
Transmission coefficient for different values of $L_{w}$, the red curve corresponds to 0.0 V, and the black curve corresponds to 0.4 V. (**a**) with $n_{d}$ fixed at 1.2$\;\times \;10^{18}$ [1/cm${}^{3}$], and (**b**) with $n_{d}$ fixed at 10$\;\times \;10^{18}$ [1/cm${}^{3}$]. The shaded area indicates the region between the bottom of the conduction band and the quasi-Fermi level at the emitter.

Figure 6 shows the transmission probability for 0.0 V red curve and for 0.4 V black curve, Figure 6a is for $n_{d}$ = 1.2$\;\times \;10^{18}$ [1/cm${}^{3}$], Figure 6b is for $n_{d}$ = 10$\;\times \;10^{18}$ [1/cm${}^{3}$]; from bottom to top, the results are indicated by increasing the width of the QW. The shaded area indicates the region between the bottom of the conduction band and the quasi-Fermi level at the emitter. As the width of the well increases, a new resonant state with higher energy and higher mean amplitude emerges for both electron densities, this state appears for $L_{w}\geq 6$ nm in the case of $n_{d}$ = 1.2$\;\times \;10^{18}$ [1/cm${}^{3}$] and for $L_{w}\geq 8$ nm in the case of $n_{d}$ = 10$\;\times \;10^{18}$ [1/cm${}^{3}$], as indicated in Figure 6a,b with the red curve. For larger $L_{w}$, the first quasi-stationary state appears closer to the bottom of the conduction band at the emitter (which corresponds to 0.0 energy), which generates the appearance of the first current peak for lower voltages; this effect is more significant in the case of lower donor density. Concerning the increase in donor density, there is a slight shift in transmission peaks toward lower energies, which translates into reaching resonance slightly faster than for the lower density. For the 0.4 V voltage, it is possible to see how the peaks have shifted, approaching the bottom of the conduction band and presenting a decrease in intensity produced by the asymmetry of the potential generated by the effects of the applied electric field.

**Figure 7:**
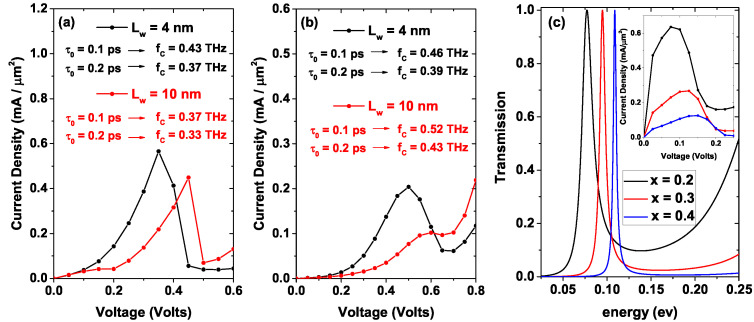
Tunneling current density for two different values of $L_{w}$ as a function of bias voltage, in (**a**) with $n_{d}$ = 1.2$\;\times \;10^{18}$ [1/cm${}^{3}$], and (**b**) with $n_{d}$ = 10$\;\times \;10^{18}$ [1/cm${}^{3}$]. Figure (**c**) shows the transmission for three different values of the Al concentration in the barriers, *x* = 0.2, 0.3, and 0.4 for a system of three regions Al${}_{x}$Ga${}_{1-x}$As/GaAs/Al${}_{x}$Ga${}_{1-x}$As. The inset shows the current density for these three systems taking $L_{w}$ = 4 nm and $L_{b}$ = 3 nm. In figures (**a**,**b**), the cut-off frequencies have been included for all the arrangements calculated (black text corresponds to $L_{w}$ = 4 nm and red text corresponds to $L_{w}$ = 10 nm) by taking two different values of $\tau_{0}$, 0.1 ps and 0.2 ps.

Figure 7 presents the tunneling current density calculated employing Equation (13) for two different values of $L_{w}$, $L_{w}=4$ nm black points and $L_{w}=10$ nm red points, as a function of the bias voltage with $L_{b}=3$ nm. In Figure 7a $n_{d}$ = 1.2 $\;\times \;10^{18}$ [1/cm${}^{3}$], in Figure 7b $n_{d}$ = 10$\;\times \;10^{18}$ [1/cm${}^{3}$]. An increase in the magnitude of the current peak is evidenced for $L_{w}=4$ nm compared to $L_{w}=10$ nm; this is due to a greater amplitude in the electron transmission probability associated with the only quasi-stationary level for the smaller well compared to the amplitude for the larger system. This is evidenced in Figure 5a comparing the mean amplitudes of the resonant states for both systems, obtaining; as a result, a higher mean amplitude for the system with lower $L_{w}$, that is, for $L_{w}=4$ nm, this behavior holds for both electron densities. Note how there are two peaks associated with the current for the system $L_{w}=10$ nm one for 0.15 V and the other for 0.45 V, reaching current density values of the order of 0.042 [mA/μm${}^{2}$] and 0.449 [mA/μm${}^{2}$], respectively. Note the difference in magnitude of these two current density peaks, and it is because the first maximum corresponds to the resonant transmission with the lower energy red peak in Figure 5, which, for non-zero voltages, when it reaches values below the quasi-Fermi level at the emitter, this peak has a very low magnitude compared to that of the second resonant peak. On the other hand, for $L_{w}=4$ nm there is only one of greater magnitude for 0.35 V, reaching a current density magnitude of 0.566 [mA/μm${}^{2}$]; this is because the larger system has two quasi-stationary states (states inside the well), while the smallest system presents only one, as evidenced in Figure 6a with the red curves. The marked difference concerning the magnitude of the current peaks associated with $L_{w}=10$ nm is due to the difference in amplitude of the quasi-stationary states, the amplitude being much smaller for the state of lower energy. For both well lengths, NDR occurs, that is, a decrease in current density from a certain limit voltage. In Figure 7b, which corresponds to a higher donor density, it is evident that the system with $L_{w}=4$ nm reaches the maximum current density faster than the system with $L_{w}=10$ nm. This is since in this system, the quasi-stationary state with the lowest energy reaches the bottom of the conduction band at the emitter with a negligible amplitude and average width compared to the second quasi-stationary state; this means that in Figure 7b, the peak presented at 0.6 V of the red curve corresponds to a current of 0.102 [mA/μm${}^{2}$] is due to the resonance generated by the second state inside the well. For the system with $L_{w}=4$ nm, the resonance with the only quasi-steady state is presented for a value of 0.5 V, which corresponds to a current density value of 0.204 [mA/μm${}^{2}$]. For both values of $L_{w}$, with $n_{d}$ = 10$\;\times \;10^{18}$ [1/cm${}^{3}$], NDR is presented. For voltages higher than 0.6 V and 0.8 V in Figure 7a and Figure 7b, respectively, there is a monotonous increasing behavior in current density due to the combination of two processes, the first being tunneling, not resonant, that is, tunneling through a single potential barrier, and the second is a probable transmission of charge carriers in regions above the potential barriers.

For comparison, Figure 7c shows the transmission for three different values of the aluminum concentration in the barriers, *x* = 0.2, 0.3, and 0.4, for a three-region system of Al${}_{x}$Ga${}_{1-x}$As/GaAs/Al${}_{x}$Ga${}_{1-x}$As. The inset shows the current density for these three systems taking $L_{w}$ = 4 nm and $L_{b}$ = 3 nm. We can note that the transmission peak width is inversely proportional to the *x*-percentage in the barrier region; this implies an increase in the system current density for the system with the lowest *x*-concentration, as can be seen in the inset of Figure 7c. On the other hand, it presents a blue shift proportional to the *x*-percentage in the barriers; this is due to the fact that when the Al percentage increases, there is an increase in the barrier heights and this displaces the quasi-stationary state towards higher energies. In Figure 7a,b the cut-off frequencies values, $f_{C}$, have been included for all the arrangements calculated (black text corresponds to $L_{w}$ = 4 nm and red text corresponds to $L_{w}$ = 10 nm) by taking two different values of the $\tau_{0}$-parameter, 0.1 ps and 0.2 ps [50]. These frequencies were calculated according to Equation (14). The highest cut-off frequency occurs for the RTD of $L_{w}$ = 10 nm and $n_{d}$ = 10$\;\times \;10^{18}$ [1/cm${}^{3}$]. Taking $\tau_{0}$ = 0.1 ps, $f_{C}=0.52$ THz. On the contrary, the lowest cut-off frequency occurs for the RTD of $L_{w}$ = 10 nm with $n_{d}$ = 1.2$\;\times \;10^{18}$ [1/cm${}^{3}$], taking $\tau_{0}$ = 0.2 ps, with a value of 0.33 THz. Note how the cut-off frequency reaches higher values for $\tau_{0}$ = 0.1 ps than for $\tau_{0}$ = 0.2 ps for both central well widths. On the other hand, the RTD with $L_{w}$ = 4 nm does not present significant changes in the cut-off frequency with the increase in carrier density $n_{d}$, the largest change is of the order of 0.03 THz for $\tau_{0}$ = 0.1 ps. For the RTD with $L_{w}$ = 10 nm, the change in cut-off frequency with increasing carrier density $n_{d}$ is more significant, reaching a maximum change of the order of 0.15 THz for $\tau_{0}$ = 0.1 ps.

**Figure 8:**
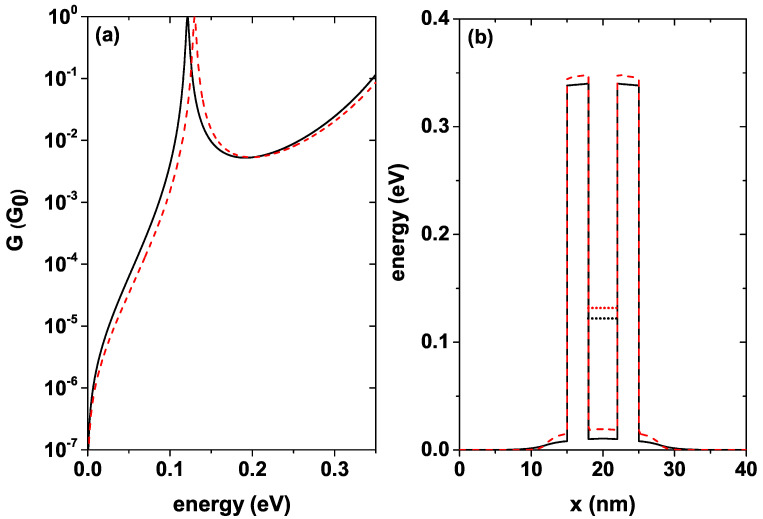
(**a**) Conductance for $L_{w}=4$ nm, for two different donor concentrations in units of $G_{0}=e^{2}/\pi \hbar^{2}$, solid black line $n_{d}$ = 1.2$\;\times \;10^{18}$ [1/cm${}^{3}$], and dashed red line $n_{d}$ = 10$\;\times \;10^{18}$ [1/cm${}^{3}$]. (**b**) Corresponding self-consistent potentials. The curves were calculated at T=5 K.

The system conductance is proportional to the electronic probability transmission. The proportionality constant is known in the literature as the conductance quantum and is given by $G_{0}=e^{2}/\pi \hbar^{2}$. Figure 8a shows the conductance as a function of the incident electron energy for a well width $L_{w}=4$ nm for T=5 K, this function is calculated by means of Equation (10), each curve corresponds to a different donor density level, the black solid curve is for $n_{d}$ = 1.2$\;\times \;10^{18}$ [1/cm${}^{3}$], and the red dashed curve is for $n_{d}$ = 10$\;\times \;10^{18}$ [1/cm${}^{3}$]. For the given value of $L_{w}$, the system presents a single resonant state; note that as the donor density increases, a blue shift occurs in the conductance peaks, and these quasi-stationary states are ordered from the system with the lowest $n_{d}$ to the system with the highest $n_{d}$, 0.1213 eV, and 0.1299 eV, respectively. It should be noted that for both curves, the resonant peak average width remains approximately independent of the donor density in the system. The intensity of the resonant peaks must reach the maximum value, that is, a 100% probability of electronic transmission when the energy of the incident electrons exactly coincides with the energy of the quasi-stationary states inside the well; this result was expected since the system is in equilibrium or equivalently without applied fields. Figure 8b shows the self-consistent potential corresponding to each donor density with which the curves in Figure 8a were calculated. Notice how the height of the central region changes with the increase of $n_{d}$, taking the quasi-stationary state towards higher energies. This figure also shows the position of the quasi-stationary states inside the well for each $n_{d}$. It must be taken into account that the conductance at the calculated temperature, that is, T=5 K, differs very little from the conductance at room temperature, which is in agreement with experimental results such as those mentioned in [53].

Table 1 presents in detail the value of the quasi-steady state corresponding to the two donor concentrations calculated, as well as their difference ΔE. The value of the potential in the center of the well and its difference for both configurations is also included. As mentioned above, the highest energy state corresponds to the system with the highest donor density. The energy difference between the states corresponding to the two concentrations is 8.6 × 10${}^{-3}$ eV, while the potential difference reaches a value slightly greater than 8.8 × 10${}^{-3}$ eV.

**Figure 9:**
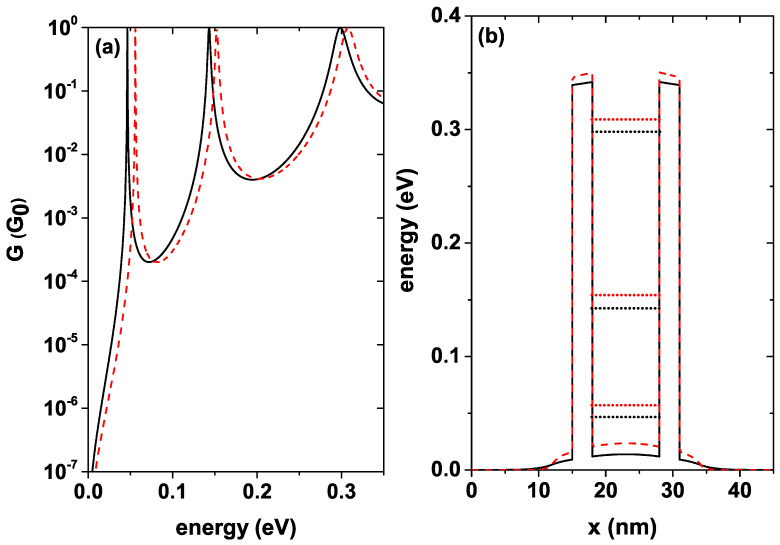
(**a**) Conductance for $L_{w}=10$ nm, for two different donor concentrations in units of $G_{0}=e^{2}/\pi \hbar^{2}$, solid black line $n_{d}$ = 1.2$\;\times \;10^{18}$ [1/cm${}^{3}$], and dashed red line $n_{d}$ = 10$\;\times \;10^{18}$ [1/cm${}^{3}$]. (**b**) Corresponding self-consistent potentials. The curves were calculated at T=5 K.

Figure 9a shows the conductance as a function of the energy of the incident electron for a well width $L_{w}=10$ nm and T=5 K, each curve corresponds to a different donor density level as in Figure 8 for $L_{w}=4$ nm. The black solid curve is for $n_{d}$ = 1.2$\;\times \;10^{18}$ [1/cm${}^{3}$], and the red dashed curve is for $n_{d}$ = 10$\;\times \;10^{18}$ [1/cm${}^{3}$]. With the increase in the well width, the number of resonant states in the system increases; this is evident by comparing Figure 8a and Figure 9a. For this greater width, the same shift behavior of the states towards higher energies occurs as the donor density increases. The two curves present a very sharp peak for the first state inside QW and two more peaks of greater amplitude for an intermediate energy state and for the state closer to the continuum.

As detailed in Table 2, the system with $n_{d}$ = 1.2$\;\times \;10^{18}$ [1/cm${}^{3}$] presents three peaks in conductance with energies of 0.046 eV, 0.143 eV, and 0.299 eV respectively, in the same way, the system with $n_{d}$ = 10$\;\times \;10^{18}$ [1/cm${}^{3}$] also presents three peaks with energies of 0.056 eV, 0.152 eV, and 0.308 eV respectively. For both configurations, there are three conductance peaks. Table 2 also shows the energy difference ΔE between each of the states corresponding to the different configurations, as well as the potential difference in the center of the well. The difference in energy becomes smaller for the highest states, that is, the states closest to the continuum are practically unchanged by the difference in donor concentration. An important conclusion is that the average width of the conductance peaks is independent of the density of donors in the system; what is modified is the position of the peaks, generating a shift towards higher energies. Figure 9b shows the self-consistent potential profile corresponding to each donor density with which the curves in Figure 9a were calculated. For this greater well width, the central region height is modified in a more significant way as compared to the depth of the smaller well width as $n_{d}$ is increased. This figure also shows the position of each of the states for the two calculated concentrations.

### Comparison with Experimental Data

**Figure 10:**
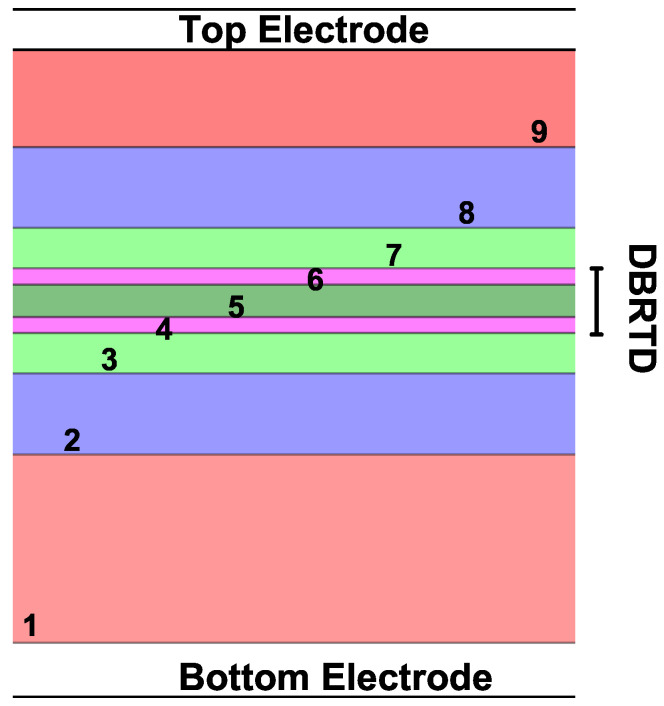
RTD structure composed of 9 layers that are expanded in detail in Table 3. DBRTD stands for Double-Barrier Resonant Tunneling Diode.

One way to test the method is through comparison with experimental results. In this subsection, a comparison is made with experimental results obtained by Muttlak et al. [54] in 2018, in which the authors presented an experimental study of InGaAs/AlAs resonant tunneling diodes designed to improve the diode characteristics by varying geometric characteristics. Figure 10 shows a diagram of the simulated device that is made up of nine layers, of which the DBRTD (Double Barrier Resonant Tunneling Diode) zone, the spacer layers that are on both sides of the DBRTD zone, and zones 1, 2, and 8, 9, which is where donors are added to the system. This arrangement of layers is connected to two electronic reservoirs that are also presented in the figure.

Table 3 shows in detail the characteristics of the materials, as well as the layer dimensions and the donor densities corresponding to those presented in Figure 10. The outer regions are composed of In${}_{0.53}$Ga${}_{0.47}$As with large dimensions compared to the central region of the device, the DBRTD region is made up of two AlAs barriers with equal widths of 1.1 nm and the QW region is In${}_{0.8}$Ga${}_{0.2}$As with a width of 3.5 nm.

**Figure 11:**
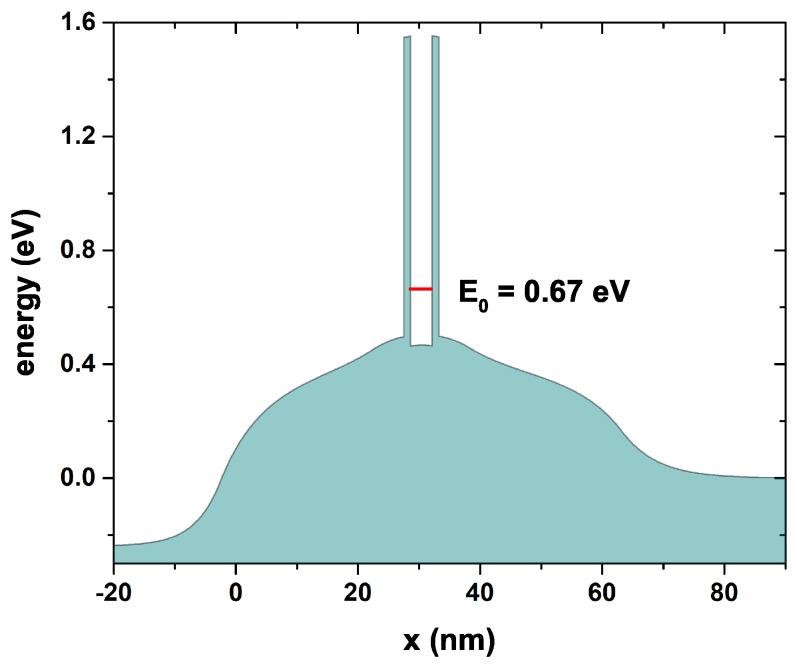
Self-consistent potential corresponding to the conduction band obtained numerically with the experimental parameters detailed in Table 3.

Figure 11 shows the self-consistent potential corresponding to the background of the conduction band obtained using the parameters presented in Table 3 at a temperature of 300 K that comes from an experimental development. The white region in the figure corresponds to the conduction band of the system, and the red segment indicates the first quasi-stationary state inside the well that has an energy of 0.67 eV and is near the bottom of the well. Note how the initially flat potential is modified considerably due to the electronic redistribution generated by the self-consistent method that considers the effect of the density of donors in the outer layers (regions 1, 2, 8, and 9). Note how the system is asymmetric concerning the center of the QW due to the asymmetry in the regions outside the DBRTD; these differences are both geometric and form the density of donors in each layer.

**Figure 12:**
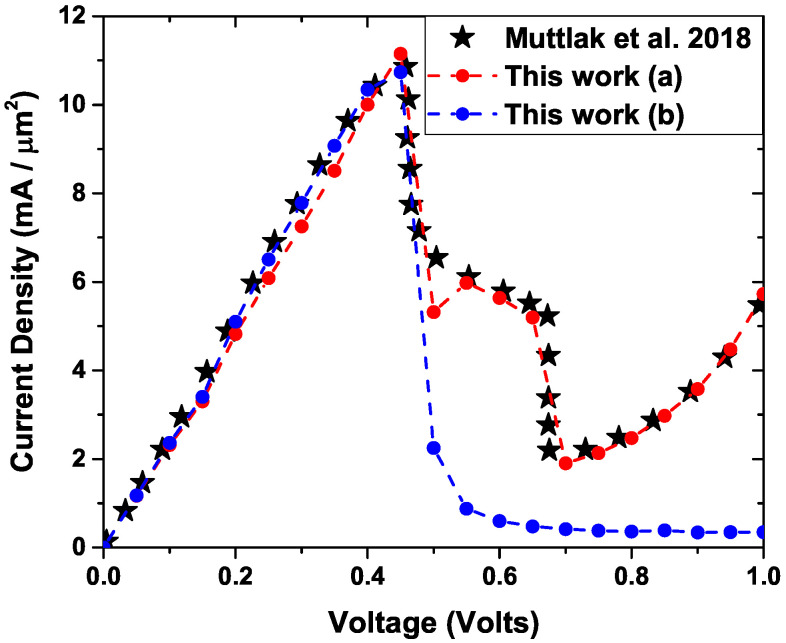
Comparison between simulated results using Equation (13) (red and blue dots) and experimental results [54] (black stars).

Figure 12 shows a comparison between the results using our model for the self-consistent calculation of the conduction band bottom profile and later use it to calculate the transmission through the Schrödinger equation in the system and finally using Equation (13) which corresponds to a Landauer approach, calculating the current density in the device. The red dots (a) correspond to the current density due to resonant tunneling, including the scattering effects simulated as additional resonances in the system, and the blue points (b) correspond to the current density obtained only by resonant tunneling, while the black stars are the experimental points. The simulation parameters that correspond to the characteristics of the materials, the dimensions, as well as the donor density are presented in Table 3 at a temperature of 300 K, which corresponds to the temperature reported on the experimental level.

In the region between 0 V and 0.46 V, there is a very good correspondence of the simulated results with the experimental ones, with a change in the current density between 0 and 10.9 [mA/μm${}^{2}$] approximately, which corresponds to the maximum value generated by the resonance between the incident electrons and the first quasi-stationary state inside the well shown in Figure 11. For values greater than 0.46 V, the simulation presents a drop in current density representing an NDR. For voltages higher than 0.5 V, the current in the system is mainly due to dispersion effects (this is evident due to the difference between the blue points and the red points in this region), due to possible impurities in the interlayer regions that can eventually contribute to electronic transport in the system. On the other hand, because the experimental temperature is 300 K, it is important to consider dispersion effects due to thermionic emission and electronic absorption of phonons, processes that can provide electrons with enough energy to tunnel through barriers and contribute to current density [55,56]. These effects are included in the model by adding three additional resonances to the simulated one at positions 1.02 eV, 1.12 eV, and 1.81 eV, respectively. These effects correspond to the red dots in Figure 12 that generate a current peak between 0.5 V and 0.7 V and exponential-like behavior for voltages higher t.

## 4. Conclusions

The wave functions, quasi-stationary states, and self-consistent potentials, among other electronic properties in a double-barrier resonant tunneling diode system based on GaAs and InGaAs, were calculated by solving the equations in each step using the finite-element method. Employing the Schrödinger equation, the probabilities of electronic transmission were calculated considering variations in geometric parameters such as the width of the central well and non-geometric parameters such as the density of donors in the layers outside the barrier region. Additionally, the system has been converged out of equilibrium to analyze the response of the internal quasi-stationary states to an external potential difference applied to the contacts, obtaining a redshift in all transmission peaks regardless of the donor density used. A way has been found to tune the system, particularly the position or quantity of quasi-stationary states inside the central well, by modifying the bias voltage, modifying the width of the central well, and modifying the density of donors in the system. Once the system was characterized through the probability of electronic transmission, the Landauer formalism was used to calculate the electric current density that circulates through the diode for different well widths and different donor densities. An important conclusion is that the first current peak is obtained for lower voltages in the case of the narrower width of the central well. On the other hand, when the donor density is lower, the current peaks reach a higher value for the simulated parameters. For the cases studied, it is possible to show NDR. For the system under study, the cut-off frequency was calculated, analyzing geometric and non-geometric variations. A maximum value of 0.52 THz has been found for the RTD of $L_{w}$ = 4 nm and $n_{d}$ = 10$\;\times \;10^{18}$ [1/cm${}^{3}$].

The conductance in the double barrier system was calculated, changing the dimensions of the well and the density of donors, obtaining multiple peaks of conductance for a width of 10 nm and a single peak for a width of 2 nm. The increase in concentration only modifies the position of the peaks, but does not change the shape of the conductance function. Finally, the theoretical procedure was applied to an experimental system reported in recent literature; this is a non-symmetric system based on InGaAs with AlAs barriers consisting of nine regions. The current density at room temperature for this system was compared, obtaining satisfactory results for calculating the position of the first resonance in the system and the magnitude of the current density at this point. Likewise, the converged parameters for the experimental comparison do not exceed 3% error compared to the same parameters reported in the literature. These results indicate that this system could be a good candidate for potential applications in various science or engineering fields.

## Figures and Tables

**Table 1:** Energy associated with the conductance peaks and potential at the center of the well and their differences ΔE for the two calculated concentrations, the data correspond to Figure 8.

$n_{d}$ ($10^{18}$ [1/cm${}^{3}$])	1.2	10	ΔE (10${}^{-3}$ eV)
*V* (eV)	0.0105	0.0193	8.8
$E_{1}$ (eV)	0.1213	0.1299	8.6

**Table 2:** Energy associated with the conductance peaks and potential at the center of the well and their differences ΔE for the two calculated concentrations, the data correspond to Figure 9.

$n_{d}$ ($10^{18}$ [1/cm${}^{3}$])	1.2	10	ΔE (10${}^{-3}$ eV)
*V* (eV)	0.0138	0.0235	9.7
$E_{1}$ (eV)	0.0463	0.0558	9.5
$E_{2}$ (eV)	0.1432	0.1523	9.1
$E_{3}$ (eV)	0.2986	0.3076	9.0

**Table 3:** Parameters corresponding to each of the layers in Figure 10.

Parameters by Layer			
**Layer**	**Material**	**Dimensions (nm)**	**Doping (** $n^{+}$ **cm** ${}^{-3}$ **)**
1	In${}_{0.53}$Ga${}_{0.47}$As	400	1 × 10${}^{19}$
2	In${}_{0.53}$Ga${}_{0.47}$As	25	3 × 10${}^{18}$
3	In${}_{0.53}$Ga${}_{0.47}$As	5	
4	AlAs	1.1	
5	In${}_{0.8}$Ga${}_{0.2}$As	3.5	
6	AlAs	1.1	
7	In${}_{0.53}$Ga${}_{0.47}$As	5	
8	In${}_{0.53}$Ga${}_{0.47}$As	25	3 × 10${}^{18}$
9	In${}_{0.53}$Ga${}_{0.47}$As	45	2 × 10${}^{19}$

## Data Availability

Not applicable.
